# Influence of Antipsychotic Drugs on Human Endogenous Retrovirus (HERV) Transcription in Brain Cells

**DOI:** 10.1371/journal.pone.0030054

**Published:** 2012-01-11

**Authors:** Olivia Diem, Marisa Schäffner, Wolfgang Seifarth, Christine Leib-Mösch

**Affiliations:** 1 Institute of Virology, Helmholtz Zentrum München, German Research Center for Environmental Health, Neuherberg, Germany; 2 III. Medizinische Klinik, UMM-Universitätsmedizin Mannheim, University of Heidelberg, Mannheim, Germany; Chiba University Center for Forensic Mental Health, Japan

## Abstract

Human endogenous retroviruses (HERVs) have been associated with various neurological and neuropsychiatric disorders. Transcripts and proteins of at least three HERV groups, HERV-W, ERV9 and HERV-K(HML-2) have been detected repeatedly in brain samples or cerebrospinal fluid of patients with schizophrenia suggesting that alterations in HERV activity may play a role in etiopathogenesis. Current therapies otherwise include neuroleptics and/or antidepressants that may induce epigenetic alterations and thus influence HERV expression. To investigate the effects of these drugs on HERV transcriptional activity, HERV expression profiles of a broad range of human brain cell lines treated with valproic acid (VPA), haloperidol, risperidone, and clozapine were analyzed using a retrovirus-specific microarray and qRT-PCR. Investigation of 52 HERV subgroups revealed upregulation of several class I and class II HERV elements by VPA in a dose-dependent manner. The strongest effect was observed on HERV-W and ERV9 groups in the human glioblastoma cell lines SK-N-SH and SK-N-MC, respectively. The transcript level of HERV-K(HML-2) elements was not influenced. Transcription of HERV-W, ERV9 and HERV-K(HML-2) taxa was further quantified in postmortem brain samples of patients with schizophrenia, bipolar disorders and a healthy control group with regard to their medication. Patients with schizophrenia showed a significantly higher HERV-W transcription associated with VPA treatment. However in case of ERV9, enhanced transcript levels could not be explained solely by VPA treatment, since a slight increase was also found in untreated patients compared to healthy controls. HERV-K(HML-2) elements appeared to be upregulated in some patients with bipolar disorders independent from medication. In conclusion, these results suggest that antipsychotic medication may contribute to increased expression of distinct HERV taxa in patients with neuropsychiatric diseases.

## Introduction

Schizophrenia is a highly complex and severe neuropsychiatric disorder with uncertain etiology. Based on data sets from family, twin, and adoption studies, as well as from epidemiological surveys, the etiopathogenesis of schizophrenia involves the interplay of polygenic influences and environmental risk factors operating on brain maturation during pregnancy [1], [2], [3], [4]. Susceptibility to environmental factors may be under genetic control and, vice versa, environmental factors can influence the genomic imprint leading to altered gene expression. Moreover, such epigenetic alterations may be vertically transferred to offspring [5]. Environmental factors such as winter-spring births, perinatal infections, household crowding, upbringing in urban areas, and pet ownership support the concept that schizophrenia could be triggered by infectious agents affecting the brains of genetically susceptible individuals [1], [6], [7], [8], [9].

Retroviruses are candidate infectious agents in CNS diseases of unknown etiology because of their neurotropism and latency (reviewed in [10], [11]). In particular human endogenous retroviruses (HERVs) have been repeatedly associated with schizophrenia and other neurological diseases (reviewed in [6]). HERVs are natural components of the human genome constituting at least 8% of human DNA. They are considered remnants of ancient germ line infections by exogenous retroviruses that have been genetically fixed and transmitted in a Mendelian fashion [12], [13]. During evolution, these elements were amplified and spread throughout the genome by repeated events of retrotransposition and/or reinfection. To date, they comprise about 500,000 elements [14]. Efficient cellular mechanisms have evolved to restrict their intracellular activities, including epigenetic mechanisms such as DNA methylation and chromatin remodeling, as well as posttranscriptional processing and RNA interference [15], [16], [17]. However, at least some members of most HERV groups were found to be still transcriptionally active in a tissue-specific manner [18], [19].

In brain samples, cerebrospinal fluid and blood of patients with schizophrenia and schizoaffective disorders elevated levels of transcripts and/or proteins from at least three HERV groups, HERV-W, ERV9 and HERV-K(HML-2), have been repeatedly detected [20], [21], [22], [23], [24], [25], [26]. This suggests failure of the cellular control mechanisms leading to activation or upregulation of distinct HERV elements in schizophrenia.

However, most schizophrenic patients had obtained antipsychotic medication for years [20], [22], [24], [26]. Even first episode patients or patients with resent-onset schizophrenia were on medication for at least one or more weeks prior study intake [22], [23], [25]. Therefore, irrespective to the question whether the observed alterations in HERV activity are causative or a consequence of the disease, a severe imponderability in appraising experimental data is the treatment of patients with neuroleptics and/or antidepressants known to influence gene expression by inducing epigenetic modifications [27], [28]. Thus, the question arises whether the overrepresentation of certain HERV transcripts in brain tissue from patients with schizophrenia may be due to the effects of drugs rather than to the disease.

To address the potential impact of medication on HERV activity we analyzed the HERV transcription pattern in a broad range of human brain cells (glioblastoma, neuroblastoma, neural stem cell lines) treated with different concentrations of valproic acid (VPA), haloperidol, risperidone, and clozapine by means of a retrovirus-specific microarray (RetroArray) and quantitative reverse transcriptase PCR (qRT-PCR). Data derived from cell culture experiments were then compared with the HERV activity in 56 postmortem brain samples with regard to medication of the respective patients.

## Results

**Figure 1:**
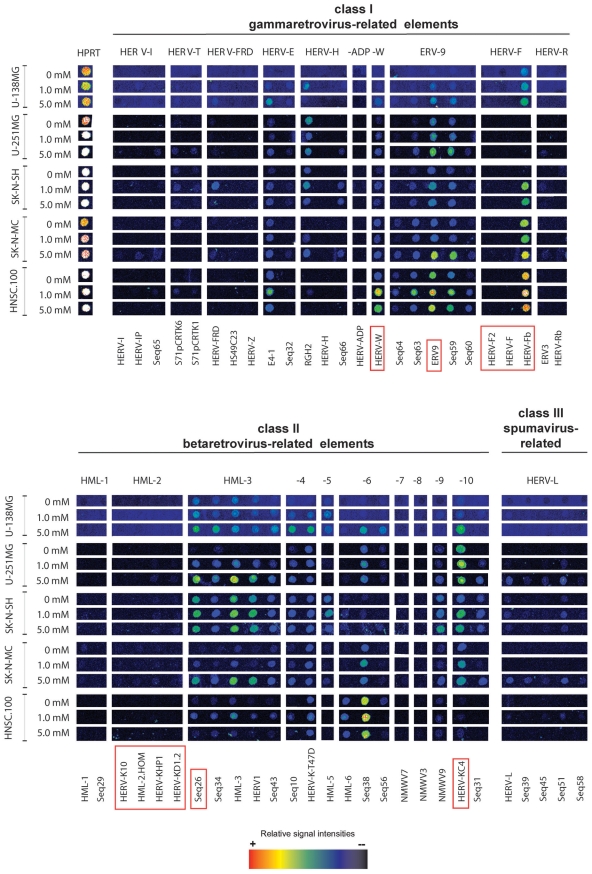
HERV transcription activity in different human brain cell lines after treatment with valproic acid. 15 representative examples of false-color microarray data sets representing untreated and treated (1 or 5 mM VPA) samples from different cell lines were aligned. For detailed information about the identity of microarray capture probes see references [19], [20]. Each positive spot on the microarray represents multiple HERV loci assigned to one subgroup of multicopy HERV elements with sufficient sequence homology that they cannot be distinguished on an individual basis. The housekeeping gene HPRT served as an internal control. Quantitative RT-PCRs were performed for a subset of six differentially active taxa (HERV-W, ERV9, HERV-F, HML-2, Seq26, and HERV-KC4 indicated by red boxes.

To investigate the influence of antipsychotic drugs on HERV expression, different human brain cell lines were selected for cell culture models including glioblastoma cells (U-138MG and U-251MG), neuroblastoma cells (SK-N-SH and SK-N-MC), and the human neural stem cell line HNSC.100. All cell lines showed nearly the same HERV core transcription profile composed of 12 HERV subgroups of class I and II HERVs that has been established previously for human brain tissue [20] (Table S1). All cell lines were treated with different doses of VPA. U-138MG, SK-N-SH, and HNSC.100 cells were additionally treated with haloperidol, risperidone, or clozapine. The drugs were used in concentrations that comply with clinically relevant doses [28] and are non-cytotoxic as determined by a MTT-assay. All further experiments were performed with RNA samples from at least two independent treatments. Alterations of HERV transcription patterns were monitored with a retrovirus-specific microarray that allows simultaneous detection of 52 representative HERV subgroups of class I (gammaretrovirus-related), class II (betaretrovirus-related), and class III (spumaretrovirus-related) HERVs [19], [29]. A representative microarray experiment of cells treated with 1 and 5 mM VPA is shown in Figure 1. Beside the brain core HERV profile defined in [20], which consists of HERV taxa with ubiquitous activity (HERV-E, HERV-F, ERV9 and HERV-K) some rarely transcribed HERV elements (members of HERV-T, HERV-FRD, HML-1, HML-2, HML-5, and HERV-L groups) were detected. A dose-dependent up-regulation of transcription activity upon treatment with VPA was observed for several class I groups (HERV-E, HERV-H, HERV-W, ERV9, HERV-F) and class II HERV groups (HML-3, HML-4, HML-6, HML-9, HML-10) (Figure 1).

Table 1 summarizes the transcriptional activity of HERV taxa after treatment of the cells with VPA, haloperidol, risperidone, or clozapine each at the highest concentration. In contrast to VPA, treatment with haloperidol or risperidone led only to a slight activation of HERV-E, RGH2 (group HERV-H), HERV-Fb (group HERV-F), HERV-W, ERV9 (group ERV9), HML-9 and HERV-KC4 (group HML-10). In case of haloperidol a dose-dependent upregulation of HERV-Fb was observed particularly in SK-N-SH cells. Clozapine showed slightly increased activities of ERV9 and HERV-KC4 (group HML-10). Remarkably, members of the HML-2 group appear not to be influenced by any medication in all cell lines according to HERV Chip data.

**Table 1:** Influence of antipsychotic medication on HERV activity in human brain cell lines.

HERV	medication													
	valproic acid					haloperidol			risperidone			clozapine		
	U138	U251	SKNSH	SKNMC	HNSC	U138	SKNSH	HNSC	U138	SKNSH	HNSC	U138	SKNSH	HNSC
**HERV-I**	−	−	−	−	−	−	−	−	−	−	−	−	−	−
**HERV-T**	−	−/+	−/0	−	−	−/0	0/+	−	−/0	−	−	−/0	0	−
**HERV-FRD**	−	−	−/0	−	−	−/0	−	−	−	−	−	−	−	−
**HERV-E**	+/0	+/0	+	+/0	+	+	+/0	0/−	0/+	0	0	0	0	0/+
**RGH2(HERV-H)**	0	0/−	0/+	0/+	0/−	0	0	0/−	−	0	+	−/0	0	0/+
**HERV-ADP**	−	−	−	−	−	−	−	−	−	−	−	−	−	−
**HERV-Fb(HERV-F)** *	++/0	−/++	++	−/+	+/0	+/0	+/++	+	+	0	0/+	+/0	0	0/+
**HERV-W** *	0/+	0/+	+/0	0	0/++	+	+	0/−	+	0	0	0	0	0/+
**HERV-R**	−	−	−	−	−	−	−	−	−	−	−	−	−	−
**ERV9** *	+	+	++/+	+/++	+	+	+	+/0	+/0	0	0	+	0	0/+
**HML-1**	−	−	0/−	−	−	−	−	−/0	−	−	−	−	−	−
**HML-2** *	−	−	−	−	−	−	−	−	−	−	−	−	−	−
**Seq26 (HML-3)** *	+/0	++/0	++/+	+/++	+/0	0	0	−/0	+/0	0	0	−	0	+/0
**HML-4**	+/0	+	+/0	0/+	0	0/+	0/+	0/−	0/+	0	0	0	0	0
**HML-5**	0	−	+	−/0	0/−	0/−	−	−	0/−	−	−	−	−	−
**HML-6**	+/0	+	+/0	0/+	+	0/+	0	0	0	0	0	0	0	0/+
**HML-7**	−	−	−	−	−	−	−	−	−	−	−	−	−	−
**HML-8**	−	−	−	−	−	−	−	−	−	−	−	−	−	−
**HML-9**	+/0	+/−	0/+	−/+	−/0	−/0	0	−/0	0	0	+	0	0	0/+
**HERV-KC4(HML-10)** *	++/0	+/++	+	0/+	+/0	+/0	0/+	0/+	+/0	+	0/+	0	+	0/+
**HERV-L**	−	−	−	−	−	−	−	−	−	−	−	−	−	+/−

Two glioblastoma cell lines (U-138MG and U-251MG), two neuroblastoma cell lines (SK-N-SH and SK-N-MC) and the human neural stem cell line HNSC.100 were treated with 5 mM VPA and compared to untreated cells. U-138MG, SK-N-SH and HNSC.100 were also treated with 10 µM haloperidol, risperidone, or clozapine. Data were obtained from at least two microarray experiments of each of two independent antipsychotic drug treatments. Varying results for the two treatments are indicated by a backslash.

− no expression; 0 no influence; + weak influence; ++ strong influence.

Abbreviations: U138 (U-138 MG), U251 (U-251 MG), SKNSH (SK-N-SH), SKNMC (SK-N-MC), HNSC (HNSC.100).

*HERV subgroups selected for quantification by qRT-PCR.

To confirm and quantify the microarray results qRT-PCR was performed for HERV taxa HERV-W, ERV9 (group ERV9), HERV-F, HML-2, Seq26 (group HML-3) and HERV-KC4 (group HML-10) (boxed in red in Figure 1 and indicated by an asterisk in Table 1). Specific primer pairs derived from the *pol* gene were used for groups HERV-W and HERV-F, subgroup-specific *pol* primers for ERV9, Seq26 and HERV-KC4 [19]; M. Vincendeau, unpublished data). For HERV-K(HML-2) group-specific primers derived from the *gag* gene were used enabling a strict discrimination to other HERV-K groups.

**Figure 2:**
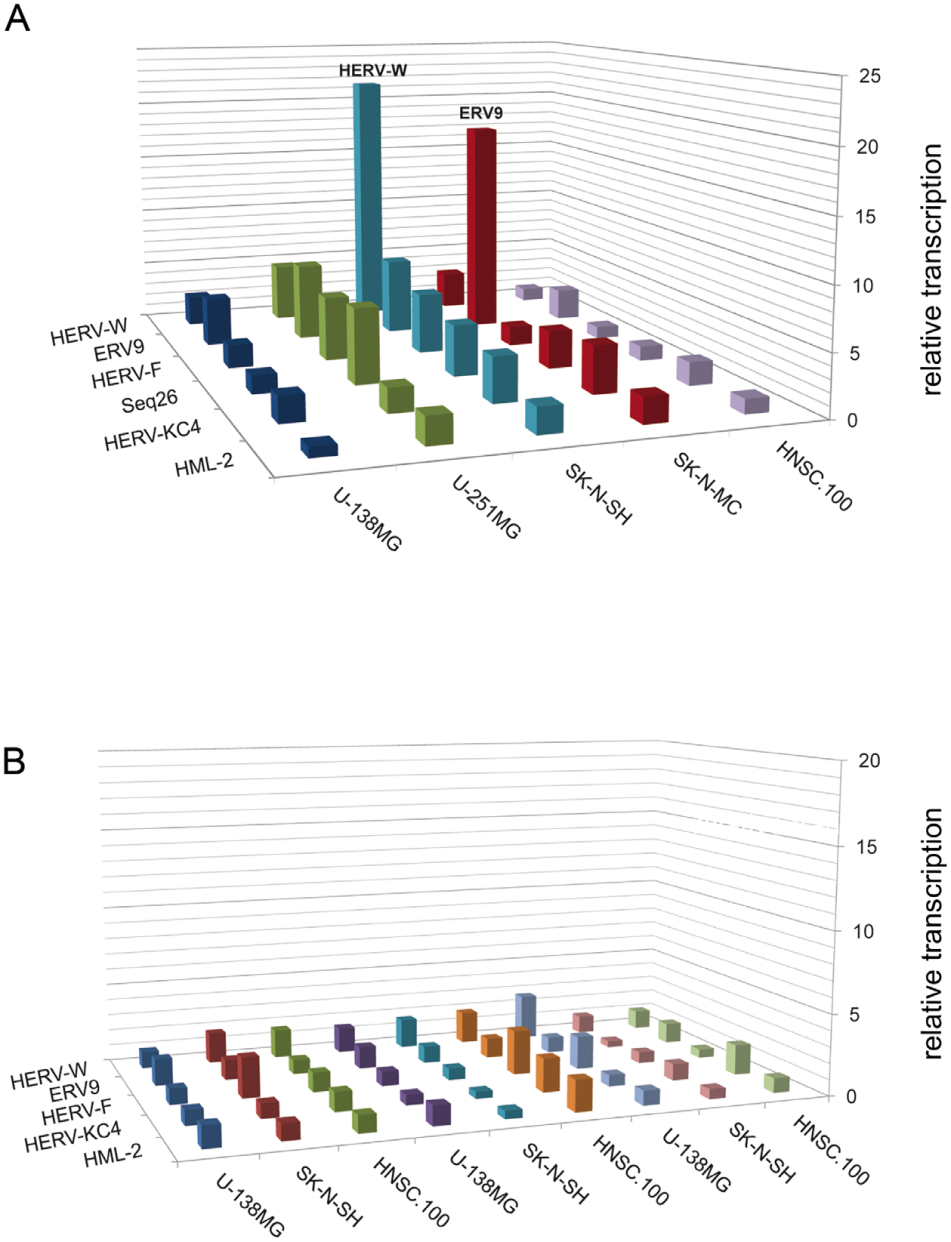
Relative transcriptional activity of selected HERV taxa in human brain cell lines. Cells were treated with (**A**) 5 mM VPA and (**B**) 10 µM haloperidol, risperidone, or clozapine, and compared to untreated cells. QRT-PCR experiments were performed on RNA samples that were obtained from two independent treatments with VPA, haloperidol, risperidone, or clozapine previously used for the retrovirus-specific microarray. Relative transcription levels were quantified according to Pfaffl et al. [41]. For amplification of HERV-K(HML-2) transcripts primers derived from the *gag* region were used. Transcription of other HERV subgroups was analyzed using *pol* specific primers overlapping with the capture probes of the microarray. The level of HERV transcripts was normalized to RPII and represents the mean value of at least triplicate qRT-PCR experiments. Numbers on the Y-axis show the fold up-regulation. A less than 2.5-fold increase of transcript levels is not considered as a significant transcriptional activation.

QRT-PCR was carried out with RNA from U-138MG, U-251MG, SK-N-SH, SK-N-MC and HNSC.100 cells treated with 1 mM and 5 mM VPA in two independent experiments. The average increase of the relative transcription of six HERV taxa at a concentration of 5 mM VPA is summarized in Figure 2 A. Detailed data are shown in Figure S1 and Table S2. In all glioblastoma and neuroblastoma cell lines, a VPA dependent upregulation of at least one HERV subgroup was observed. In U-251MG cells HERV-W, ERV9, HERV-F as well as Seq26 were found to be upregulated up to six fold compared to untreated cells. A dramatic alteration of HERV activity was observed in neuroblastoma cells. HERV-W transcription was increased on average 22-fold in SK-N-SH cells, and ERV9 18-fold in SK-N-MC cells after treatment with VPA. In the neural stem cell line HNSC.100 no significant upregulation was observed, but a very high VPA dependent activity of the cell cycle control gene p21 (data not shown). No significant influence of VPA on the transcription activity of the HERV-K(HML-2) group was found.

In contrast to VPA, the typical neuroleptic haloperidol as well as the atypical neuroleptics risperidone and clozapine showed nearly no effect on HERV transcription (Figure 2 B). Only HERV-F appeared to be slightly upregulated in SK-N-SH after treatment with haloperidol and in HNSC.100 cells after addition of risperidone; HERV-W in U-138MG cells after treatment with clozapine.

**Figure 3:**
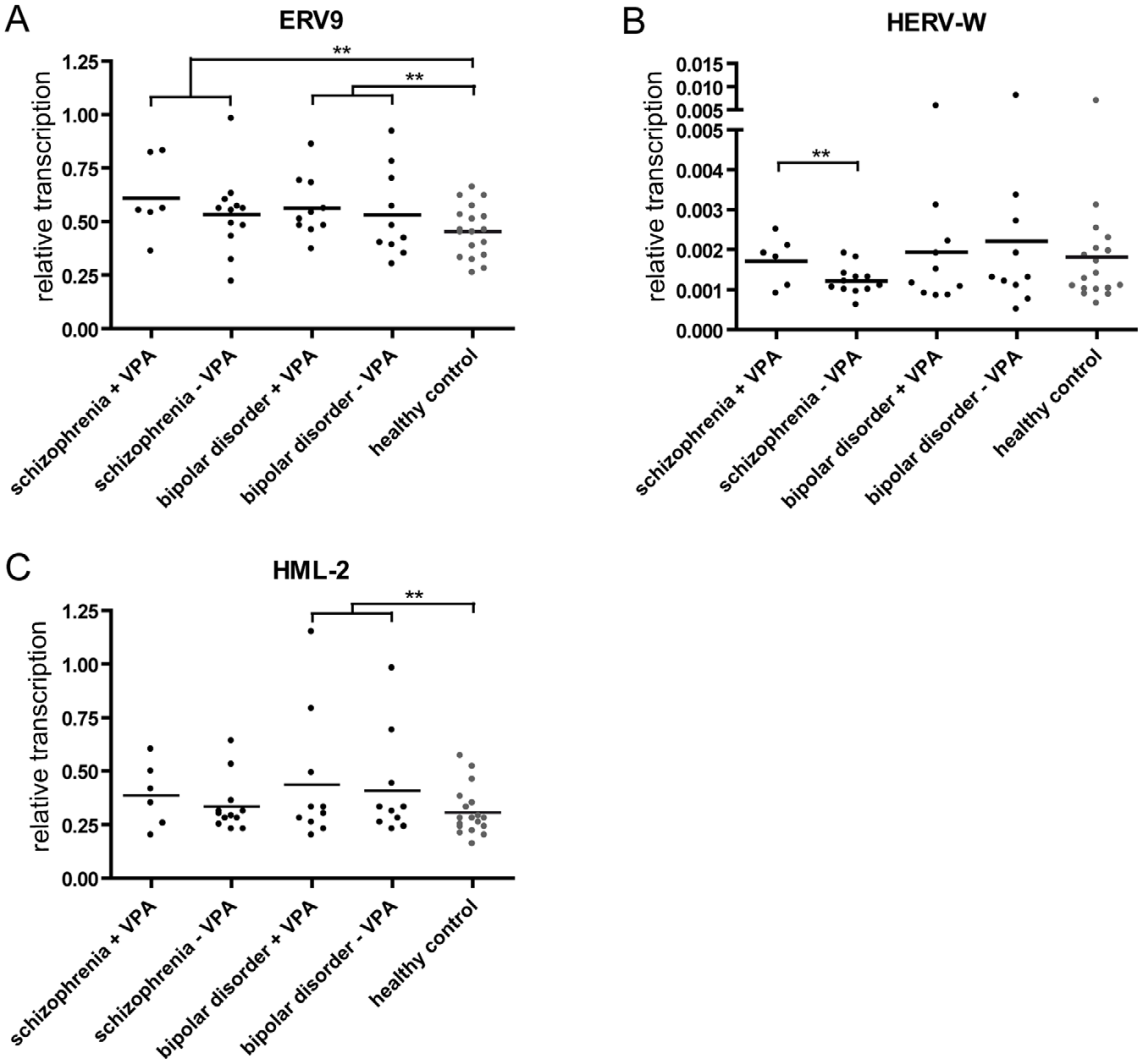
Quantification of HERV transcription in 56 postmortem brain samples by qRT-PCR. Transcriptional activities of ERV9 (**A**), HERV-W (**B**), and HML-2 (**C**) were analyzed in a subset of 18 schizophrenia-derived brain samples and 20 samples of patients with bipolar disorders with regard to VPA treatment, and compared to 18 healthy individuals. The level of HERV transcripts in each sample was normalized to RPII levels and represents the mean value of at least triplicate experiments. Significant deviation between patient groups according to the Student's t-test is denoted for each HERV taxon by * p≤0.05, ** p≤0.01, *** p≤0.001).

The HERV groups HERV-W, ERV9 and HERV-K(HML-2) have been repeatedly associated with schizophrenia. As VPA showed a significant influence on HERV-W and ERV9 transcription but not on HERV-K(HML-2) in cell culture models, we further investigated the transcription of ERV9, HERV-W, and HERV-K(HML-2) in postmortem brain samples of patients with schizophrenia and bipolar disorders with regard to their antipsychotic medication (Figure 3). QRT-PCR was performed on RNA samples from the Stanley Array Collection [8] previously used for microarray experiments [20]. 18 samples of patients with schizophrenia, 20 samples of patients with bipolar disorders, and 18 healthy controls were examined.

A slight but not significant increase of ERV9 and HERV-K(HML-2) transcription was observed in both patient groups under VPA treatment. A significant up-regulation of HERV-W transcription was detected in schizophrenic patients obtaining VPA. Independent of the medication, transcription of ERV9 was found significantly increased in patients with schizophrenia and bipolar disorders compared to the healthy control group. Likewise, transcript levels of HERV-K(HML-2) elements were elevated in patients with bipolar disorders with and without VPA treatment. The transcription of HERV-W was not significantly higher in both patient groups.

To exclude that alcohol or drug abuse may influence the data, we further grouped the patients according to drug or alcohol abuse. No significant influence of alcohol or drug consumption on HERV-W, ERV9 and HERV-K(HML-2) transcription was observed (Figure S2).

## Discussion

HERVs, in particular HERV-W, ERV9, and HERV-K(HML-2) have been associated with schizophrenia and other neurological diseases [20], [21], [22], [24], [25]; reviewed in [6]. Elevated levels of HERV transcripts and/or proteins detected in brain samples, plasma or cerebrospinal fluid of patients might be etiologic factors or a consequence of the disease. Moreover, they could be indicators for epigenetic changes induced by medication influencing the epigenetic environment.

Using a retrovirus-specific microarray as well as qRT-PCR we have investigated the influence of antipsychotic drugs on HERV transcription in five brain derived cell lines including neural stem cells, gliobastoma and neuroblastoma cells. No or only slight effects were observed for haloperidol, risperidone, and clozapine. In contrast, VPA increased the transcription of many HERVs, including members of groups ERV9 and HERV-W. VPA is a histone deacetylase inhibitor and thus may cause chromatin remodeling around HERV promoters leading to enhanced expression. Previous investigations have shown that VPA induces chromatin modifications and, in combination with other antipsychotics, alteration of DNA methylation patterns in patients with schizophrenia and bipolar disorders [30], [31], [32]. Interestingly, in our study the effects of VPA appear to be cell type-dependent and are predominantly observed in both neuroblastoma cell lines. SK-N-SH and SK-N-MC differ in several features, for example in expression levels of dopamine-beta-hydroxylase [33], suggesting a differential epigenetic background. This might explain that transcription of two different HERV groups, HERV-W and ERV9, is highly increased by VPA in SK-N-SH and SK-N-MC, respectively.

Transcription of group HERV-K(HML-2) is not significantly influenced by any drugs in all cell lines investigated. In a previous study, expression profiling of a broad range of HERVs in brain samples from patients with schizophrenia and bipolar disorders using the retrovirus-specific microarray revealed a significant overrepresentation of HERV-K(HML-2) transcripts in both patient groups compared to healthy controls [20]. To verify these data, and to reassess a possible influence of antipsychotic drugs, we used the same patient material for qRT-PCR. Sorting the data according to patient medication we observed a bias to an increased transcriptional activity of ERV9 and HERV-W in brain tissue of schizophrenic patients treated with VPA in comparison to untreated patients. In addition to the effect of VPA, a slight elevation of ERV9 transcripts was observed in both patient groups compared with healthy controls. Independent of the medication, a significant upregulation of HERV-K(HML-2) transcription was found in some patients with bipolar disorders. These data suggest that transcriptional activation of certain retroviral elements might be associated with the disease at least in some cases. However, these data should be interpreted with caution because many confounding factors, demographic and clinical variables, may conceal the outcome of the experiments [27] Such imponderabilities may also explain differential findings of recently published studies [20], [21], [22], [24], [25]. Drug abuse and alcohol, the parameters with the most influence [26], were therefore analyzed in this study, but did not show relevant differences.

Taken together, our data suggest a complex regulation of HERV activity in human brain cells. Differential HERV expression in patients may depend on environmental factors including epigenetic drugs, as well as pathologic conditions. Moreover, individual variation of epigenetic patterns may play a role. Most HERVs constitute multicopy families, e. g. HERV-K(HML-2) comprises around 60 different loci per haploid human genome, 16 of which are differentially transcribed in human brain [34]. Groups HERV-W and ERV9 represent about 40 and 300 proviral copies, respectively. Each provirus of one HERV group might be differentially expressed under varying epigenetic environments. This could explain inconsistent results obtained in different experimental settings. An interesting hypothesis is that transcriptional activation of many defective HERV copies may interfere with few protein coding HERVs [26]. This could for example be provoked by antisense HERV transcripts expressed from adjacent cellular promoters [34] or bidirectional HERV promoters [35]. Such a self-regulating mechanism may constitute a complex network of HERV transcriptional control that has helped HERVs to escape purifying selection during evolution.

## Materials and Methods

### Human brain RNA samples

Total RNA samples derived from the prefrontal cortex (Brodmann's area 46) of 12 patients with schizophrenia, 20 patients with bipolar disorders and 18 healthy controls (The Stanley Array Collection) were kindly provided by the Stanley Medical Research Institute, Bethesda, MD. The Stanley Medical Research Institute reassures that in all cases the next of kin gave permission in writing to examine the brains for research purposes. A detailed statement about the Stanley Brain Collection can be obtained from the Institute's website at www.stanleyresearch.org.

### Cell lines and drug treatment

The human glioblastoma cell lines U-138MG (HTB-16) and U-251MG (HTB-17), the human neuroblastoma cell lines SK-N-SH (HTB-11) and SK-N-MC (HTB-10), and the human neural stem cell line HNSC.100 [36] were purchased from ATCC or authenticated by the German Collection of Microorganisms and Cell Cultures (DSMZ, Braunschweig, Germany) and cultured as recommended. Cell culture media were supplemented with 10% fetal calf serum and penicillin-streptomycin at a concentration of 100 µg/ml. All cells were routinely tested for mycoplasma contamination using LookOut Mycoplasma PCR Detection Kit, Sigma. Cells were treated with 1 and 5 mM valproic acid (VPA) or 0.1, 1 and 10 µM haloperidol, risperidone, or clozapine (Sigma-Aldrich, Steinheim, Germany) for 24 h. U-251MG cells were additionally treated with 1 and 5 mM VPA for 72 h. The concentrations were non-cytotoxic as determined by a colorimetric MTT assay in 96 well plates as described [37]. As controls untreated cells of the same passage were cultivated in parallel with each experiment.

### RNA Preparation, DNAse treatment, and cDNA synthesis

Total RNA was isolated from cultured cells using the RNeasy Kit according to manufacturer's instructions (Qiagen, Hilden, Germany). To exclude DNA contamination, RNA samples were treated with 100 units/µg RNase-free DNase using the RQ1 DNase Kit (Promega, Mannheim, Germany) and tested by PCR with mixed oligonucleotide primers [19]. Only DNA-negative RNA samples (1–2 µg) were subsequently transcribed using SuperScript II First-Strand Synthesis according to the manufacturer's instructions (Invitrogen, Karlsruhe, Germany).

### Retrovirus-specific microarray (RetroArray)

Hybridization probe synthesis and labeling by MOP-PCR, as well as DNA chip preparation, hybridization, and postprocessing of retrovirus-specific microarrays were performed as described previously [18], [19], [20]. Hybridized microarrays were scanned using an Affymetrix Scanner GMS 418 (laser power 100%, gain 50–60%) and false color mapping was used for image visualization.

### Quantification of HERV transcripts by qRT-PCR

For the amplification of *pol*(RT) sequences, specific primers for groups HERV-W (forward primer: TGAGTCAATTCTCATACCTG, reverse primer: AGTTAAGAGTTCTTGGGTGG) and HERV-F (forward primer: CCTCCAGTCACAACAACTC, reverse primer: TATTGAAGAAGGCGGCTGG) were used. Subgroup-specific primers were employed for ERV9 (group ERV9) (forward primer: CCTCAACTGTTTTAATGTCTTAGGGCGAGG, reverse primer: CCCTCATCTGTTTGGTCAGGCCC), Seq26 (group HML-3) (forward primer: CTGCAGCCTGCTAAGCG, reverse primer: CACTGTGAAAATTTTTTACGAG), and HERV-KC4 (group HML-10) (forward primer: GAATCTCTTCTAATTTGAACCTTTTGAGG, reverse primer: CCCACAGTTTGTCAAACTTTTGTAGGC) [19]; M. Vincendeau, unpublished data). For group HERV-K(HML-2), *gag* specific primers were used for quantification (forward primer: GGCCATCAGAGTCTAAACCACG, reverse primer: CTGACTTTCTGGGGGTGGCCG). As a control for VPA treatment p21 was quantified as described [38].

QRT-PCR was performed with the Roche LightCycler 480 System, using LC480 DNA Master SYBR Green and the standard LightCycler protocol (Roche Diagnostics, Mannheim). A 10-minute initial denaturation step at 95°C was followed by 50 amplification cycles of 10 s at 95°C, 5 s at 60°C, and 10 s at 72°C. Melting curves were generated for the final PCR products by decreasing the temperature to 65°C for 15 s followed by an increase in temperature to 95°C. Fluorescence was measured at 0.2°C increments. RNA-Polymerase II (RPII) transcripts were analyzed as internal standards [39]. ΔC_T_-values were calculated as follows: *C_T_*(RPII)-*C*_T_(HERV element), and were normalized to RPII levels. The x-fold induction of HERV transcription in treated cells was calculated by the 2^−ΔΔCT^ method [40], with values normalized to RPII and relative to transcription in non-treated cells. The relative HERV transcription ratio of postmortem samples was calculated from the qRT-PCR efficiencies and the crossing point deviations of the target gene versus the housekeeping gene RPII [41]. Quantitative RT-PCR experiments for each gene were performed at least in triplicate.

## Supporting Information

Figure S1 **Quantification of HERV transcription in human brain cell lines by qRT-PCR.** Transcriptional activities of HERV-W (**A**), HML-2 (**B**), ERV9 (group ERV9) (**C**), Seq26 (group HML-3) (**D**), HERV-F (**E**), and HERV-KC4 (group HML-10) (**F**) were analyzed in cell lines after treatment with 1 and 5 mM VPA in comparison to untreated cells. Relative transcription levels were quantified according to the method of Pfaffl et al. [41]. For amplification of HERV-K(HML-2) transcripts primers derived from the *gag* region were used. Transcription of other HERV subgroups was analyzed using *pol* specific primers overlapping with the capture probes of the microarray. The level of HERV transcripts in each sample was normalized to RPII levels and represents the mean value of at least triplicate experiments of two independent treatments.
(TIF)

Figure S2 **HERV transcriptional activity in 56 postmortem brain samples with regard to heavy alcohol or drug use analyzed by qRT-PCR.** Transcriptional activities of ERV9 (**A**), HERV-W (**B**), and HML-2 (**C**) in brain samples of patients with schizophrenia, bipolar disorders and healthy individuals (Figure 3) were grouped according to heavy alcohol or drug use irrespective of the disease and VPA treatment. No significant differences in HERV transcription were observed.
(TIF)

Table S1 **Baseline HERV activity in different human brain cell lines.**
(PDF)

Table S2 **Relative transcriptional activity of six selected HERV taxa in human brain cell lines after treatment with 5 mM VPA, 10 µM haloperidol, risperidone, or clozapine.**
(PDF)
