# Supplementary figures and images for: Influence of Antipsychotic Drugs on Human Endogenous Retrovirus (HERV) Transcription in Brain Cells
Source: PLoS One. 2012 Jan 11;7(1):e30054. doi: 10.1371/journal.pone.0030054 (PMC3256206; doi:10.1371/journal.pone.0030054)

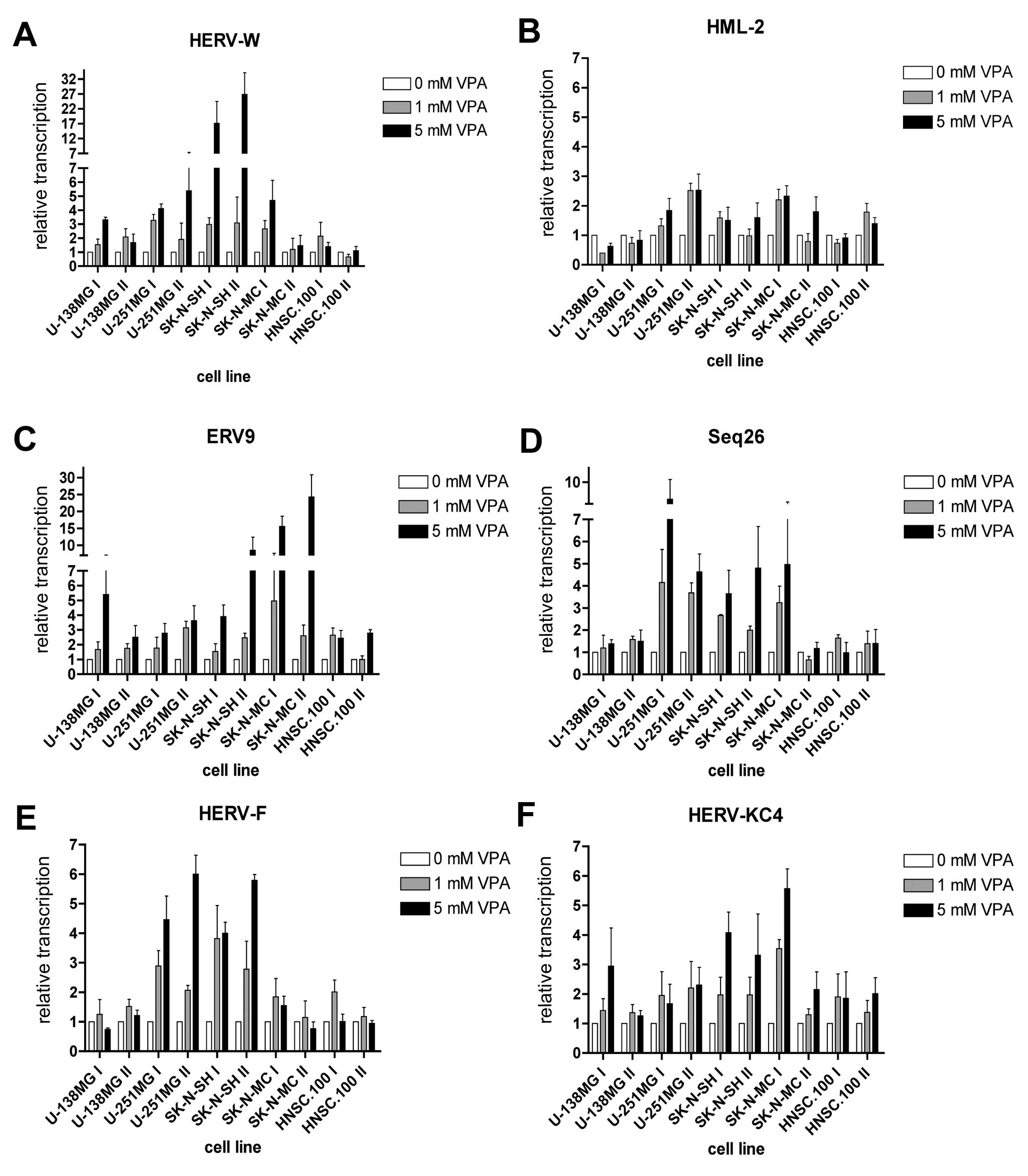

Supplement: Figure S1 — Quantification of HERV transcription in human brain cell lines by qRT-PCR. Transcriptional activities of HERV-W (A), HML-2 (B), ERV9 (group ERV9) (C), Seq26 (group HML-3) (D), HERV-F (E), and HERV-KC4 (group HML-10) (F) were analyzed in cell lines after treatment with 1 and 5 mM VPA in comparison to untreated cells. Relative transcription levels were quantified according to the method of Pfaffl et al. [41]. For amplification of HERV-K(HML-2) transcripts primers derived from the gag region were used. Transcription of other HERV subgroups was analyzed using pol specific primers overlapping with the capture probes of the microarray. The level of HERV transcripts in each sample was normalized to RPII levels and represents the mean value of at least triplicate experiments of two independent treatments. (TIF) [file pone.0030054.s001.tif]

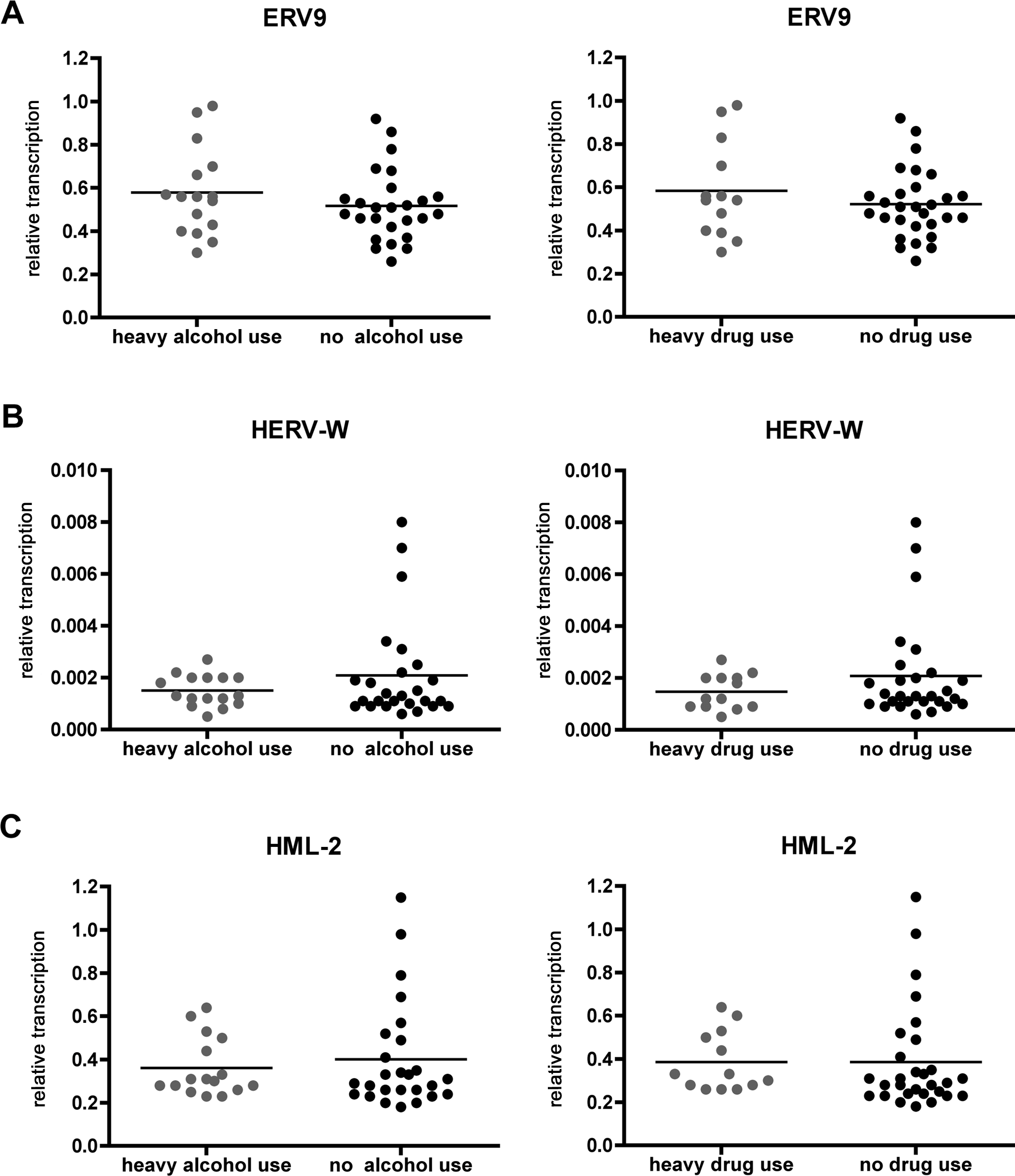

Supplement: Figure S2 — HERV transcriptional activity in 56 postmortem brain samples with regard to heavy alcohol or drug use analyzed by qRT-PCR. Transcriptional activities of ERV9 (A), HERV-W (B), and HML-2 (C) in brain samples of patients with schizophrenia, bipolar disorders and healthy individuals (Figure 3) were grouped according to heavy alcohol or drug use irrespective of the disease and VPA treatment. No significant differences in HERV transcription were observed. (TIF) [file pone.0030054.s002.tif]
