# Supplementary material for: Influence of Antipsychotic Drugs on Human Endogenous Retrovirus (HERV) Transcription in Brain Cells
Source: PLoS One. 2012 Jan 11;7(1):e30054. doi: 10.1371/journal.pone.0030054 (PMC3256206; doi:10.1371/journal.pone.0030054)
Supplement: Table S1 — Baseline HERV activity in different human brain cell lines. (PDF) [file pone.0030054.s003.pdf]

Table S1

Baseline HERV activity in different human brain cell lines.

	HERV groups (subgroups)	U-138 MG	U-251 MG	SK-N-SH	SK-N-MC	HNSC.100
class I	HERV-I	-	-	-	-	-
	HERV-T	-	-	+	-	-
	HERV-FRD	-	-	-	-	-
	HERV-E *	+/-	+	+	+	+
	RGH2 (HERV-H)	-	+	+	-	-
	HERV-ADP	-	-	-	-	-
	HERV-Fb (HERV-F) *	+	+/-	+/-	+	+
	HERV-W	+/-	+/-	+	+	+
	HERV-R	-	-	-	-	-
	ERV9 *	+	+	+	+	+
class II	HML-1	-	-	-	-	-
	HML-2 *	-	-	-	-	-
	Seq26 (HML-3)	+	-	+	+	+
	HML-4 *	+/-	+	+	+	+
	HML-5	+	-	+	-	-
	HML-6 *	+/-	+	+	+	+
	HML-7	-	-	-	-	-
	HML-8	-	-	-	-	-
	HML-9 *	+/-	+	+	+	+
	HERV-KC4 (HML-10) *	+	+	+	+	+/-
class III	HERV-L	-	-	+	-	-

\+ active in both experiments
+/- active in one experiment

Data were obtained from at least two microarray experiments of two individually grown cell cultures.

* (sub-)groups representing the retroviral core activity in human brain [20].
