# Supplementary material for: Influence of Antipsychotic Drugs on Human Endogenous Retrovirus (HERV) Transcription in Brain Cells
Source: PLoS One. 2012 Jan 11;7(1):e30054. doi: 10.1371/journal.pone.0030054 (PMC3256206; doi:10.1371/journal.pone.0030054)
Supplement: Table S2 — Relative transcriptional activity of six selected HERV taxa in human brain cell lines after treatment with 5 mM VPA, 10 µM haloperidol, risperidone, or clozapine. (PDF) [file pone.0030054.s004.pdf]

Table S2

Relative transcriptional activity of six selected HERV taxa in human brain cell lines after treatment with 5 mM VPA, 10 µM haloperidol, risperidone, or clozapine.

		HERV-W	ERV9	HERV-F	HERV-KC4	HML-2	Seq26
5 mM VPA	U-138MG	2.5	4.0	1.9	2.1	0.8	1.5
	U-251MG	4.8	6.4	5.3	2.0	2.2	6.2
	SK-N-SH	21.6	6.3	5.0	3.7	2.1	4.2
	SK-N-MC	3.1	17.9	1.6	3.9	2.1	3.1
	HNSC.100	1.1	2.6	1.0	1.9	1.2	1.2
10 µM haloperidol	U-138MG	1.0	1.7	1.0	0.9	1.3	n.t.
	SK-N-SH	1.9	1.2	2.5	0.9	1.0	n.t.
	HNSC.100	1.8	0.9	1.2	1.2	1.1	n.t.
10 µM risperidone	U-138MG	1.6	1.4	0.9	0.6	1.2	n.t.
	SK-N-SH	1.8	1.1	0.7	0.4	0.5	n.t.
	HNSC.100	2.0	1.3	2.8	2.1	1.9	n.t.
10 µM clozapin	U-138MG	2.8	1.0	2.1	0.7	0.9	n.t.
	SK-N-SH	1.1	0.4	0.7	1.0	0.6	n.t.
	HNSC.100	1.2	1.3	0.6	1.9	0.9	n.t.

Values represent the mean transcript levels (fold up-regulation compared to untreated cells) of two independent drug treatments. QRT-PCR experiments were performed in triplicate.
